# Assessment of quality of life and psycho-emotional burden in pregnant women in Greece

**DOI:** 10.18332/ejm/145963

**Published:** 2022-03-22

**Authors:** Maria Saridi, Aikaterini Toska, Dimitra Latsou, Maria-Anna Chondropoulou, Aikaterini Matsioula, Pavlos Sarafis

**Affiliations:** 1 School of Social Sciences, Hellenic Open University, Patras, Greece; 2 Department of Social and Educational Policy, University of Peloponnese, Corinth, Greece; 3 Hellenic Liver Patients’ Association ‘Prometheus’, Athens, Greece; 4 Department of Nursing, Faculty of Health Sciences, Cyprus University of Technology, Limassol, Cyprus

**Keywords:** quality of life, pregnancy, depression symptoms, Greece

## Abstract

**INTRODUCTION:**

Many changes occur in a woman’s body during pregnancy. These changes (biological, chemical, hormonal, anatomical) can make a pregnant woman both physically and mentally vulnerable. Thus, the aim of this study is to evaluate the quality of life (QoL) in association with depression symptoms in pregnancy.

**METHODS:**

A cross-sectional study was conducted in 123 pregnant women who visited one of the largest obstetrics and gynecology centers in Greece. The Edinburgh Postnatal Depression Scale (EPDS) was used to measure depression symptoms and World Health Organization Quality of Life instrument to evaluate quality of life. The collected data were organized with the SPSS software, version 25.

**RESULTS:**

The results showed that 15.5% of pregnant women were at an increased risk of developing depression symptoms; 91% of the women declared that their QoL was good/very good, whereas 92.7% was very satisfied with their health status. Depression symptoms seem to be positively correlated with the low household income, unpleasant event during pregnancy, and the trimester of pregnancy. Additionally, unmarried women, an unpleasant event during pregnancy and the second trimester of pregnancy proved to be negatively associated with the quality of life. Women without a risk of depression had better QοL than women who were at risk of depression symptoms.

**CONCLUSIONS:**

It is important to evaluate the QοL of women during pregnancy with the aim of good prenatal health. The organization of the necessary interventions for mothers’ health and their newborns are also of vital importance.

## INTRODUCTION

Pregnancy is one of the most important events in a woman’s life and is often considered a period of excitement, expectation, and change^1^. However, pregnancy is a condition that most often causes stress in women. During pregnancy, many changes occur in future mothers’ bodies, such as biochemical, hormonal, and anatomical, which are not controlled by the women, making them mentally and spiritually vulnerable^2^. Even in a normal pregnancy, these changes can alter a woman’s ability to perform her daily roles, affecting her quality of life (QoL) and her mental health^3^.

Depressive symptoms are especially evident in women in the developing world, maybe because pregnancy is a high-risk period for the mother’s life^4^. Depression during pregnancy negatively impacts maternal and child health, and is associated with unhealthy behaviors, inadequate prenatal care, and poorer maternal–fetal bonding^5^. Moreover, the onset of depression during pregnancy is a prognostic factor for the onset of postpartum depression^6^. Studies show that depression in early pregnancy and the third trimester of pregnancy is higher than after the childbirth period^7,8^.

The experience of pregnancy is individual and depends on various factors and situations which affect the general health and QoL of future mothers. Women with poor QoL may feel out of control of childbirth, increasing their stress levels^7^. Several studies have shown that pregnant women have a lower QoL, reporting poorer social functioning and reduced activity, as well as lower bodily function^8,9^. Higher QoL was strongly linked with pregnant women’s sociodemographic factors, such as the absence of economic difficulties, a high educational level^10,11^ and younger women^12^. Moreover, medical and obstetrical characteristics indicate poor quality of life, such as adverse medical history and obesity, primiparity^13,14^ and experience of infertility^15^.

Assessing the quality of life in pregnancy is particularly important in prevention and treatment and the development of maternal and neonatal care planning policies^3,16^. Counselling and support by an interdisciplinary team will detect the pregnant woman’s needs and intervene to solve the problems that arise early.

Even though Greece has one of the lowest rates of depression symptoms (4.7%) in the general population compared to the European Union average (6.6%)^17^, few related studies have been performed in Greece to assess it in pregnant women. Thus, this study aims to evaluate the QoL in association with depression symptoms in pregnancy. It is expected that the poor QoL will be correlated with depression symptoms and women with low socioeconomic profiles may be at risk of these.

## METHODS

### Study design and sample

This study is a descriptive cross-sectional study that took place at one of the largest obstetrics and gynecology centers in Greece. The duration of this study was from March to April 2018. The study population consisted of a convenience sample of pregnant women in the first, second and third trimesters of pregnancy who visited the clinic for a regular check-up. The inclusion criteria in the study were women who knew the Greek language and were aged >18 years. A total of 127 questionnaires were distributed, of which 123 were returned fully completed. The questionnaires were distributed when pregnant women visited the center for regular examination while in the waiting room.

### Questionnaires and scales

An anonymous self-administered questionnaire was used. The first part contained questions regarding the sociodemographic characteristics of the sample. The second part included questions about the obstetric history of the women and information about pregnancy such as desired pregnancy, experience of fertility, trimester of pregnancy, miscarriage, abortion, obstetric complications or unpleasant events during the pregnancy, and history of psychological problems. The third part included the Edinburgh Postnatal Depression Scale (EPDS) questionnaire, which investigates depression in pregnant women^18,19^. EPDS consists of 10 questions based on a 4-point Likert scale and graded depending on the severity or duration of each symptom (3 is the most severe symptom and the maximum score was 30). Participants completed the Greek version, and according to the authors, the cut-off score of EPDS estimated at 8.5 as the best one for screening for minor, moderate and severe depression and 12.5 for major depression^20^. The fourth part contained the World Health Organization Quality of Life (WHOQOL-BREF) instrument that comprises 24 items which measure the following broad domains: physical health (activities of daily living, dependence on medicinal substances and medical aids, energy and fatigue, mobility, pain and discomfort, sleep and rest, work capacity), psychological health (bodily image and appearance, negative feelings, positive feelings, self-esteem, spirituality/religion/personal beliefs thinking, learning, memory and concentration), social relationships (personal relationships, social support, sexual activity) and environment (financial resources, freedom, physical safety and security, health and social care accessibility and quality, home environment, opportunities for acquiring new information and skills, participation in and opportunities for recreation/leisure activities, physical environment, transport). There are also two separate questions evaluating an individual’s total QoL and satisfaction of health status, and their mean estimates of overall QoL and general health^21^. The WHOQOL-BREF assessed the individual’s perceptions of their health and well-being over the previous two weeks. The questions follow a Likert 5-point scale, where one represents ‘disagree’ or ‘not at all’ and five means ‘completely agree’ or ‘extremely’. A higher score indicates a better QoL. Women completed the Greek version^22^.

### Statistical analysis

The collected data were organized with the SPSS software, version 25. Descriptive analyses were performed, including frequencies, percentages and means. Cronbach’s alpha (internal consistency index) was used to estimate the reliability of the WHOQOL-BREF and EPDS questionnaire. The differences between nominal variables were found with Pearson’s chi-squared test. According to the Kolmogorov-Smirnov test, variables did not follow a normal distribution, so non-parametric tests were chosen. Mann-Whitney U Test and Kruskal-Wallis Test were used to investigate the differences between participants’ characteristics and their QoL or the risk of depression symptoms. Spearman correlation coefficient was used to determine the association between the WHOQOL-BREF and EPDS questionnaire dimensions. In this study, the level of significance for all analyses was set at p<0.05.

## RESULTS

Table 1 presents the demographic characteristics and obstetrics history of the sample. The majority belonged to the age group 28–37 years (66.7%), 91.9% were Greek, 73.1% completed university or a Master’s/ PhD degree, 87.8% were married, and 52.8% of the women stated that their household income was <1500 €. Regarding the obstetrics history of participants, 60.2% reported a planned pregnancy. For 58.5% of the women this was their first pregnancy, 35% was in the first trimester and an equal percentage in the third trimester. A history of at least one miscarriage and abortion was reported by 27.6% and 12.2%, respectively, and obstetric complications in this pregnancy were reported by 17%. Most of the respondents (89.5%) did not report a history of psychological problems. In comparison, 8.1% stated that they had experienced an unpleasant event during pregnancy, such as a divorce or death/illness of a close person.

**Table 1:** Sociodemographic characteristics and obstetrics history of the sample

*Characteristics*	*n*	*%*
**Age** (years)		
18–27	15	12.2
28–37	82	66.7
38–47	26	21.1
**Nationality**		
Greek	113	91.9
Other	10	8.1
**Educational level**		
Secondary school	4	3.3
High school	29	23.6
University	56	45.5
Master’s/PhD	34	27.6
**Married**		
No	15	12.2
Yes	108	87.8
**Household income** (€)*		
<1000	24	19.9
1000–1500	33	27.3
1500–2000	32	26.5
2000–2500	14	11.5
>2500	18	14.8
**Pregnancy**		
Planned	74	60.2
Not planned	49	39.8
**Experience of fertility**		
No	72	58.5
Yes	51	41.5
**Trimester of pregnancy**		
1st trimester	43	35.0
2nd trimester	37	30.0
3rd trimester	43	35.0
**Miscarriage**		
No	89	72.4
Yes	34	27.6
**Abortion**		
No	108	87.8
Yes	15	12.2
**Obstetric complications during this pregnancy**		
No	102	83
Yes	21	17
**History of psychological problems**		
No	110	89.5
Yes	13	10.5
**Unpleasant event during pregnancy**		
No	113	91.9
Yes	10	8.1

* Missing values for two participants.

As far as the depression symptoms in pregnant women, 9.8% had experienced minor, moderate and severe depression symptoms (mean: 10.5) and 5.7% major depression symptoms (mean: 14.6); 15.5% (n=19) had an increased risk of developing depression symptoms than 84.5% of women with no depression (mean: 6.52). The reliability for the EPDS questionnaire in this study was α=0.87. The EPDS questionnaire results are presented analytically in Table 2. Regarding depression symptoms, an increased risk was significantly related to household income. Furthermore, 26.3% of women who experienced an unpleasant event during pregnancy were at a higher risk of developing depression. Notably, 29.7% of the sample in the second trimester appeared to have depression symptoms, in contrast with lower percentages of those in the first or third trimester. Table 3 presents significant differences in EPDS with sociodemographic characteristics and obstetrics history of the sample.

**Table 2:** EPDS questionnaire results

*Over the last 7 days*	*n*	*%*
**I could laugh and see the pleasant side of things**		
As always	97	78.9
Not so much anymore	21	17.1
Certainly not so much now	4	3.3
Not at all	1	0.8
**I was looking forward to things happening**		
As always	98	79.7
Not so much now as before	19	15.4
Certainly, less now than I used to	4	3.3
Not at all	2	1.6
**I blamed myself when something went wrong**		
No, never	41	33.3
Not so often	44	35.8
Yes, sometimes	34	27.6
Yes, most of the time	4	3.3
**I was anxious or sad for no reason**		
Not at all	21	17.1
Very rarely	34	27.6
Yes, sometimes	58	47.2
Yes, very often	10	8.1
**I was afraid and panicked for no reason**		
No, never	35	28.5
Not so often	47	38.2
Yes, sometimes	37	30.1
Yes, quite often	4	3.3
**I was disturbed by the situations**		
No, I do as well as ever	40	32.5
No, I often cope quite well	58	46.8
Yes, sometimes I could not cope as always	24	19.4
Yes, most of the times I am not at all able to cope	1	0.8
**I was so sad that I had difficulty sleeping**		
Not at all	78	63.4
Not so often	29	23.6
Yes, sometimes	14	11.4
Yes, most of the time	2	1.6
**I felt distressed or miserable**		
Not at all	62	50.4
Not so often	53	43.1
Yes, sometimes	8	6.5
Yes, most of the time	0	0.0
**I was so sad that I cried**		
Not at all	75	61.0
Only occasionally	41	33.3
Yes, quite often	7	5.7
Yes, most of the time	0	0.0
**I thought to do harm to myself**		
Never	118	96.0
Almost never	4	3.2
Sometimes	1	0.8
Yes, quite often	0	0.0

**Table 3:** Significant differences of EPDS and WHOQOL-BREF score with sociodemographic characteristics and obstetrics history of the sample

	*No depression symptoms %*	*Risk of developing depression symptoms %*	*p*	*WHOQOL-BREF score*	*p*
**Household income** (€)					
<1500	21.1	78.9	0.032		
≥1500	41.1	58.9			
**Unpleasant event during pregnancy**					
Yes	73.2	26.3	0.008	17.12	0.042
No	95.2	4.8		15.80	
**Pregnancy trimester**					
1st	93	7	0.013	17.20	0.040
2nd	70.3	29.7		16.18	
3rd	88.4	11.6		17.50	
**Married**					
No				16.00	0.018
Yes				17.20	

**Figure 1:**
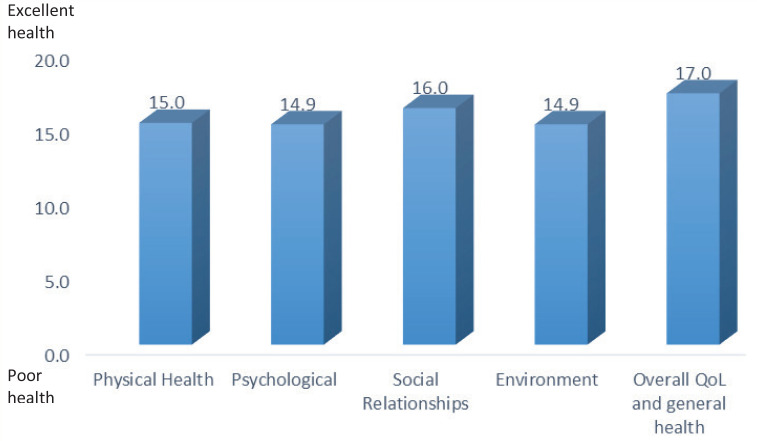
Dimensions of WHOQOL-BREF showing a good QoL of the sample

Moreover, 91% of women declared that their quality of life was good/very good, 7.3% neither poor nor good, and 1.6% very poor. None of the samples scored QoL poor; 92.7% were satisfied/very satisfied with their health status, and 7.3% answered neither satisfied nor dissatisfied. None of the women stated very dissatisfied or fairly dissatisfied. All the dimensions of WHOQOL-BREF, physical health, psychological, social relationships, environment, and overall QoL and general health, showed a good quality of life of the sample (Figure 1). The reliability for the WHOQOL-BREF questionnaire in this study was α=0.82. The mean of overall quality of life and general health was significantly lower for women who had experienced an unpleasant event during pregnancy than for those who had not an unpleasant experience. Moreover, married women reported a higher quality of life than unmarried women. The study participants in the first or third trimester scored higher in the quality-of-life scale in comparison with those in the second trimester. All the above statistically significant differences are presented in Table 3.

All dimensions of the WHOQOL-BREF score were negatively correlated with the EPDS score, which means that women with a higher risk for depression symptoms had lower quality of life. More specifically, physical health was moderately correlated with the presence of depression symptoms; however, psychological, social relationships and environment were weakly correlated (Table 4).

**Table 4:** Correlation between WHOQOL-BREF score and EPDS score

	*EPDS score r (p)*
Physical health	-0.416 (0.001)
Psychological	-0.248 (0.006)
Social relationships	-0.314 (0.001)
Environment	-0.317 (0.001)
Overall QoL and general health	-0.252 (0.005)

## DISCUSSION

Our study showed that the majority of the sample had a planned pregnancy and no previous experience of infertility. A low percentage of women faced miscarriage, abortion, obstetric complications, or unpleasant events. History of psychological problems were reported only by 10%. Significantly, the distribution of the sample in the pregnancy trimesters was approximately the same. Moreover, only 19 women were at risk of developing depression symptoms. Low-income women, who experienced an unpleasant event during pregnancy and those in the second trimester appeared to be more at risk of developing depression symptoms. The sample reported a good QoL and was very satisfied. However, better QoL was found in women who had not experienced an unpleasant event, were married, and went through the first or third trimester. Finally, women with a higher risk for depression symptoms had lower quality of life.

The prevalence of depression in the general population is estimated at 12%, however, this rate is higher in people with a prior history of major depressive disorder and in those with a history of postpartum depression^23^. In this study, 15.5% of pregnant women were at an increased risk of developing depression symptoms, which represents a significant proportion. The estimated rate of depression symptoms during pregnancy ranges from 7–15% in economically developed countries and from 19–25% in poorer countries^24,25^. In Greece, the rate of pregnant women with a history of recurrent mental disorder is high, reaching about 50%^26^. In a previous study in a public hospital in Greece, in a sample of 163 pregnant women, one-third was depressed and almost half of those were anxious^27^.

Τhe factors that appeared to affect women’s depression symptoms in this study were the income, the trimester of pregnancy, and an unpleasant event during pregnancy. More specifically, women with medium-high socioeconomic status had lower depression symptoms. This result is in accordance with other studies where the rate of depression was higher mainly due to socioeconomic factors such as poverty, unemployment, and low level of education^28-30^. However, a significant reason why only 19 women of our sample experienced depression symptoms may be that the majority of the sample was highly educated and with middle to high household income. Moreover, the proportion of depressed women was double that of non-depressed women in the second trimester of pregnancy. In our study, the pregnant women who had experienced an unpleasant event during pregnancy seem to be at an increased risk of developing depression symptoms. Similarly, Bunevicius et al.^31^ found that stressful life events were associated with depressive disorder through all trimesters of pregnancy.

In terms of physical and psychological health, social relationships and environment, the rates in our study corresponded to a good level of QοL in all these areas. These findings are consistent with other studies and highlight that the quality of life during pregnancy has proven to be very good or excellent, although they used different general questionnaires, which nevertheless measure general status and quality of health^16,32^. However, well-educated women, with a middle to high household income as our sample, stated good QoL according to Elsenbruch et al.^33^. Moreover, the second trimester of pregnancy is associated with a lower QoL compared to the other two trimesters in the present study. A reason may be that pregnant women in this trimester limited their daily activities, which have an impact on their physical health and social relationships. Also, women are expecting the highest diagnostic accuracy of fetal development, thus stress is increased. As far as marital status, Lagadec et al.^34^ declared that women had better quality of life if they were married, had family and friends. This finding is in agreement with ours, where married women stated higher quality of life.

### Limitations

There are some limitations in this study. The first limitation is that a self-reported questionnaire was used during waiting time at the clinic, hence social desirability bias may affect our results. The second is that this study was cross-sectional, and women were not followed through the whole pregnancy. Moreover, the study was conducted in only one private obstetrics, gynecology and surgery center in Attica, thus our results cannot be generalized across the country. Further longitudinal studies are needed on broader and more representative samples of women that could yield valid and reliable findings that could lead to significant physical exercise-related actions and policies.

## CONCLUSIONS

Changes during pregnancy can significantly affect the QοL of pregnant women and increase the depression symptoms rates. The monitoring of physical and mental health throughout pregnancy, as well as the training of women in their new role, by an integrated team of health professionals, are necessary priorities in the planning of health policy in Greece.

## Data Availability

The data supporting this research are available from the authors on reasonable request.
